# Metabolic, Cardiovascular, and Stress Biomarker Adaptations to Breath-Hold Training in a National-Level Swimmer: A Signal-Generating Single-Case Study

**DOI:** 10.3390/jfmk11020213

**Published:** 2026-05-28

**Authors:** Gabriella D’Orsi, Paride Vasco, Raffaella R. R. Marzovillo, Natalia Forte, Giulia Scioscia, Giuseppe Cartagena, Luigi A. Marinaccio, Maria L. Torquato, Giuseppe Cibelli, Anna A. Valenzano

**Affiliations:** 1 Department of Clinical and Experimental Medicine, University of Foggia, 71100 Foggia, Italy; raffaella.marzovillo@unifg.it (R.R.R.M.); natalia_forte.591095@unifg.it (N.F.); giuseppe.cibelli@unifg.it (G.C.); anna.valenzano@unifg.it (A.A.V.); 2 Department of Humanities Education and Sport, Pegaso University, 80143 Naples, Italy; paride.vasco@unipegaso.it; 3 Department of Medical and Surgical Science, University of Foggia, 71100 Foggia, Italy; giulia.scioscia@unifg.it; 4 Sport Medicine Unit, Policlinic of Foggia, 71100 Foggia, Italy; giuseppe_cartagena.555937@unifg.it (G.C.); luigi.marinaccio@unifg.it (L.A.M.); marialuigiatorquato@gmail.com (M.L.T.)

**Keywords:** breath-hold training, lactate, heart rate, salivary biomarkers, swimming performance

## Abstract

**Background**: Breath-hold training (BHT) has emerged as a novel strategy to enhance metabolic efficiency and autonomic resilience in national-level athletes. This signal-generating single-case study examined physiological and neuroendocrine adaptations to an eight-week BHT program in a nationally ranked competitive swimmer. **Methods**: A national-level 23-year-old female freestyle sprinter (50 m best time = 26.59 s; 100 m = 60.40 s) completed three weekly BHT sessions integrated into her regular training. Pre- and post-intervention assessments included an incremental Mader cycling test with measurements of blood lactate ($\left[{\mathrm{La}}^{-}\right]$), heart rate (HR), salivary cortisol (sCort), and salivary alpha-amylase (sAA). Blood chemistry and pulmonary function, including diffusing capacity of the lung for carbon monoxide (DLCO), were also evaluated. **Results**: Post intervention, the athlete demonstrated reduced $\left[{\mathrm{La}}^{-}\right]$ and HR at all workloads, a 20 W increase in power at 4 mmol·L^−1^ $\left[{\mathrm{La}}^{-}\right]$, and an elevated final workload achieved during the Mader test. Salivary stress biomarkers showed blunted responses with significant reductions in area under the curve and large effect sizes. These changes were observed under standardized pre-analytical conditions and individualized training adjustments. **Conclusions**: This study highlights coordinated improvements in metabolic, cardiovascular, and stress regulation mechanisms following BHT in a swimmer with verified national-level performance benchmarks. BHT, when applied in sport-specific contexts, may serve as an effective adjunct to high-performance training.

## 1. Introduction

Breath-hold training (BHT) has gained attention as a versatile and potent physiological stimulus capable of eliciting cardiorespiratory, metabolic, and autonomic adaptations relevant to both performance and health. Operationally, BHT involves repeated bouts of voluntary apnea, which can be performed at rest (static) or during exercise (dynamic), typically executed at varying lung volumes to induce intermittent states of hypoxia and hypercapnia [1]. In sport, particularly among swimmers and freedivers, BHT is leveraged to improve oxygen utilization, delay metabolic acidosis, and reduce reliance on anaerobic glycolysis during high-intensity efforts [2,3].

Mechanistically, voluntary breath-holding combines hypoxic and hypercapnic stress, stimulating the peripheral chemoreflex and modulating ventilatory and cardiovascular responses [4]. Repeated exposures to intermittent hypoxia enhances mitochondrial efficiency, increases buffering capacity, and contributes to autonomic balance [5,6]. Recent systematic reviews emphasize that the outcomes of BHT are strongly dependent on protocol design—apnea duration, recovery intervals, exercise modality, and athlete profile—underscoring the need for context-specific applications [7,8].

In competitive swimming, BHT integrated into training has been associated with improved repeat sprint ability, sprint endurance, and stroke efficiency [9,10]. Recent reviews on BHT in athletes have highlighted that apnea performed under dynamic, sport-specific conditions may yield stronger physiological responses and greater transfer to competitive performance than static, passive apnea [8].

Beyond physical performance, BHT also interacts with the hypothalamic–pituitary–adrenal (HPA) axis and sympathetic–adrenal–medullary system (SAM), both of which are sensitive to physical exertion and psychological stress. Salivary biomarkers such as cortisol (sCort) and alpha-amylase (sAA) provide a non-invasive means to evaluate these systems [11,12]. Yet, despite their relevance, these markers are seldom studied in the context of BHT, especially under ecological, real-world training conditions. This is a notable gap, given that national-level athletes often train at the limits of physiological tolerance, where precise monitoring of stress modulation and recovery may influence both acute performance and long-term adaptation [13,14].

Furthermore, responses to BHT vary widely between individuals, shaped by baseline ventilatory control, fitness level, genetic predispositions, and prior hypoxic exposure [7,15]. Acknowledging these interindividual factors is essential, since tailoring apnea loads and recovery intervals to individual tolerance appears to enhance both safety and effectiveness. Accordingly, the present single-case study aimed to evaluate whether an eight-week, individualized BHT program would induce measurable cardiorespiratory, metabolic, and neuroendocrine adaptations in a competitive female swimmer. Specifically, we hypothesized that the intervention would (a) reduce blood lactate ($\left[{\mathrm{La}}^{-}\right]$) and heart rate (HR) at matched workloads, (b) increase power output at the 4 mmol·L^−1^ lactate threshold (W4) and the final workload achieved, and (c) blunt salivary cortisol (sCort) and alpha-amylase (sAA) responses across an incremental test. Based on prior work in apnea and intermittent hypoxia [7,8,16] and on biomarker studies in sport [14,17], we anticipated coordinated adaptations under standardized conditions. We further anticipated that careful individualization of the apnea dose would enhance tolerability and the magnitude of adaptation [7,15]. Given the signal-generating purpose of this study, the integration of $\left[{\mathrm{La}}^{-}\right]$, HR, cardiopulmonary exercise testing (CPET), pulmonary diffusing capacity (DLCO), and salivary biomarkers represents a monitoring-oriented framework intended to identify plausible response patterns and generate mechanistic hypotheses for individualized training management.

## 2. Materials and Methods

### 2.1. Participant Characteristics and Screening Procedures

A signal-generating single-subject case study was implemented, involving a 23-year-old female swimmer (see Table 1 for detailed anthropometric, hematological, and biochemical characteristics) competing at the National Level in short-distance freestyle events (50 m and 100 m).

Athletic level was classified as Level 3 (National Level) according to the standardized performance classification model for swimming research proposed by Ruiz-Navarro et al. [18]. This classification appropriately reflects her regular participation in national championships, competitive times, and systematic involvement in structured high-performance training programs. At the time of the study, the athlete was actively training with Circolo Canottieri Aniene (Rome, Italy), completing a minimum of 13 sessions per week with a total swim volume exceeding 20 km. Inclusion criteria comprised prior experience with incremental exercise testing, possession of a current medical certificate for competitive swimming and freediving, and the absence of cardiovascular, respiratory, or metabolic disorders. The athlete had not engaged in altitude or hypoxic training in the previous month and was free from any medications or injuries. Written informed consent was obtained in accordance with the Declaration of Helsinki, and the study protocol was approved by the local institutional ethics committee. Anthropometric data were collected during the pre-test session. Body height was measured using a wall-mounted stadiometer, and body mass was assessed with a calibrated mechanical scale. Body composition was evaluated using bioelectrical impedance analysis (BIA 101, Akern Srl, Florence, Italy), providing estimates of fat-free mass, fat mass, total body water, resistance, reactance, and phase angle. Hematological and blood chemistry parameters—including hemoglobin, hematocrit, leukocyte subpopulations, C-reactive protein, and thyroid function markers—were obtained via venipuncture and analyzed by the accredited clinical laboratory of the Policlinic of Foggia (Italy).

### 2.2. BHT Intervention

The athlete completed an eight-week BHT program integrated into her existing swim training regimen. The program consisted of three supervised sessions per week, combining dry-land static apneas with in-water dynamic apneas. The BHT program was implemented as an “add-on” intervention, integrated into the athlete’s regular training schedule without substituting any pre-existing swimming sessions, thereby increasing the total weekly training volume.

The dry-land component consisted of three sets of five maximal voluntary apneas performed at total lung capacity (TLC). Each set was preceded by 2–3 submaximal preparatory breath-holds to facilitate the dive response and prolong the easy-going phase. During maximal efforts, the athlete was instructed to reach the “struggle phase”; as recorded in the training diary, the onset of involuntary breathing maneuvers (IBMs) typically occurred around the 60–70% mark of the total apnea duration. Each apnea was separated by two minutes of passive recovery, with a longer five-minute transition between sets. To ensure progression, the total breath-hold volume was increased by 5% every two weeks, provided the athlete reported a Rating of Perceived Exertion (RPE) below 7/10. Safety was prioritized during all sessions. In-water dynamic apneas were conducted under one-to-one supervision by a certified coach. Pre-apnea hyperventilation was strictly prohibited to mitigate the risk of hypoxic blackout, and the athlete was instructed to terminate any apnea upon the first sign of discomfort or following the planned “struggle phase” benchmarks.

The dynamic water component consisted of eight 50 m freestyle repetitions performed at approximately 70% of the athlete’s maximal HR, with controlled breathing intervals. These progressed weekly from every five strokes to every nine strokes (hypoxic swimming). Training load was increased over time by lengthening the apneic distance (from 15 m to 25 m of underwater dolphin kick per lap), shortening recovery intervals (from 60 s to 30 s), or slightly increasing swim speed while maintaining the designated breathing pattern. All sessions were supervised by certified swimming and freediving coaches and conducted in compliance with international safety guidelines for apnea [19]. Adjustments to the program were made weekly based on perceived exertion, HR recovery, and diary-reported tolerance, ensuring the training remained effective yet sustainable. This specific focus on minimizing breathing frequency aimed to reduce the kinematic disruptions and hydrodynamic drag associated with the breathing action, which can significantly impact sprinting velocity [20,21]. A detailed week-by-week description of the 8-week training protocol, including specific sets, recovery times, and progression rules, is provided in the Supplementary Material (File S1).

### 2.3. Mader Incremental Cycling Test

To assess changes in exercise tolerance and lactate dynamics, the athlete completed a Mader-type incremental cycling test at baseline and post intervention. Testing was performed on a calibrated cycle ergometer (ERG 911 Plus, Schiller AG, Baar, Switzerland) with 5 min stages increasing by 30 W per stage. The test began at a workload individualized from cardiopulmonary exercise test (CPET) results and continued until volitional exhaustion or cadence dropped below 60 rpm. Pedal cadence was maintained between 80 and 90 rpm throughout. At the end of each stage, capillary blood samples (10 μL) were collected from the earlobe for lactate analysis using a Lactate Scout 4 portable analyzer (EKF Diagnostics, Barleben, Germany). HR was continuously recorded using a Polar H10 chest strap (Polar Electro Oy, Kempele, Finland) synchronized with the ergometer’s software. HR data were averaged over the final 30 s of each stage. The W4 was determined as the workload corresponding to 4 mmol·L^−1^ $\left[{\mathrm{La}}^{-}\right]$, following the method of Mader and Heck [22].

### 2.4. Pulmonary Function Evaluation (Spirometry and DLCO)

Pulmonary function was assessed using the MasterScreen PFT system (Erich Jaeger GmbH, Würzburg, Germany), which performed both standard spirometry and single-breath diffusing capacity ($DLCO_{sb}$) measurements in accordance with ATS/ERS recommendations [23]. For spirometry, key measures included forced vital capacity (FVC), forced expiratory volume in 1 s (FEV1), and mid-expiratory flows [24]. Diffusion capacity testing involved inhalation of a gas mixture containing a low concentration of carbon monoxide and an inert tracer, followed by a 10 s breath-hold and exhalation. DLCO, alveolar volume (VA), and transfer coefficient (KCO = DLCO/VA) were calculated. At least two acceptable maneuvers meeting the American Thoracic Society and European Respiratory Society (ATS/ERS) repeatability criteria (i.e., difference ≤2 mL/min/mmHg or 10%) were obtained. Values were reported both in absolute terms and as percent predicted, accounting for age, sex, height, and hemoglobin concentration. Calibration of the system was performed daily.

### 2.5. Cardiopulmonary Exercise Assessment (CPET)

Cardiopulmonary responses to exercise were assessed before and after the BHT intervention via CPET, using the Cardiovit CS-200 Office ErgoSpiro system (Schiller AG, Baar, Switzerland). The assessment was performed on an electronically braked cycle ergometer using a discontinuous incremental protocol. Following a 3 min warm-up at 50 W, the workload was increased by 25 W every 3 min until volitional exhaustion, allowing for $\left[{\mathrm{La}}^{-}\right]$ sampling during the final 30 s of each stage. The test was conducted under breath-by-breath gas exchange analysis, following ATS/ERS guidelines [23]. Given that a clear plateau in oxygen uptake is not always observable in incremental protocols involving non-cycling-specialized athletes, parameters were defined as peak values (${\mathrm{VO}}_{2\mathrm{peak}}$ and ${\mathrm{VO}}_{2\mathrm{peak}/\mathrm{kg}}$) rather than maximal oxygen uptake (${\mathrm{VO}}_{2\max}$), representing the highest 30 s average achieved during the final stage. Other parameters included oxygen pulse (${\mathrm{VO}}_{2}$/HR), HR response, ventilatory equivalents, $VE/{\mathrm{VCO}}_{2}$ slope, and oxygen uptake efficiency slope (OUES). The athlete was tested under standardized pre-analytical conditions (e.g., fasting state, controlled hydration, consistent time of day, and matched menstrual cycle phase) and was instructed to avoid caffeine, alcohol, and strenuous exercise for at least 12 h prior.

### 2.6. Salivary Biomarker Collection and Analysis

To assess neuroendocrine responses, sCort and sAA were measured at baseline, immediately post exercise, and during recovery. To account for the diurnal rhythm of cortisol, all samples were collected between 08:30 and 10:30 AM. Saliva samples were collected using Salivette^®^ devices (Sarstedt AG, Nümbrecht, Germany) at three specific time points: 15 min before the start of the session (baseline), immediately upon cessation of the exercise protocol, and after 30 min of passive recovery. Participants performed a 10 min water rinse before each collection to remove potential contaminants. To minimize hormonal-related variability, the participant’s menstrual cycle phase was determined using the retrospective calendar method (tracking the previous three months) combined with daily basal body temperature monitoring. Both PRE and POST testing sessions were strictly scheduled during the early follicular phase (days 3 ± 1) to ensure a consistent hormonal milieu characterized by low estrogen and progesterone concentrations. Samples were centrifuged at 3000 rpm for 10 min at 4 °C and stored at −80 °C until analysis. sCort and sAA concentrations were measured in duplicate using enzyme-linked immunosorbent assays (ELISAs) provided by Salimetrics LLC (State College, PA, USA). Absorbance was read at 450 nm for sCort and 405 nm for sAA using a PowerWave XS plate reader (BioTek Instruments Inc., Winooski, VT, USA). Results are presented as the mean ± standard deviation of duplicate assays.

### 2.7. Data Processing and Analysis

The primary outcome variables included $\left[{\mathrm{La}}^{-}\right]$ concentration, HR, sCort, and sAA. Given the single-subject design, the analysis focused on descriptive and hypothesis-generating observations rather than population-level inference. Results are presented as absolute values and percent change (Δ%) from baseline. To account for measurement variability, standard deviations (SDs) are reported with specific meanings: for HR, SD reflects beat-to-beat variability during the final 30 s of an exercise stage; for salivary biomarkers, SD represents analytical variability (technical error) derived from duplicate assays.

The interpretation of changes prioritized whether the observed shifts exceeded expected day-to-day biological variability and technical error of measurement (TEM) reported in the literature for similar populations. For hormonal markers, the area under the curve with respect to ground ($AUC_{g}$) was calculated to quantify the integrated response.

## 3. Results

### 3.1. Participant Characteristics and Baseline Screening

The athlete was a highly trained national-level swimmer with established competitive benchmarks in short-distance freestyle events (50 m best: 26.6 s; 100 m best: 60.4 s in a 50 m pool), as reported in Table 2.

### 3.2. BHT Protocol Adherence and Individualization

The athlete completed all prescribed BHT sessions with 100% adherence. Dry static apnea times increased from 72 ± 6 s (week 1) to 98 ± 8 s (week 8), while inter-apnea recovery intervals were shortened from 2:00 to 1:20 min. In dynamic swimming apneas, the breath-hold pattern was adjusted from every 5 strokes to every 9, with a 6% increase in submaximal swimming speed. The athlete reported exertion levels between 5 and 6 on the CR10 scale, with no adverse effects recorded.

### 3.3. Functional and Physiological Outcomes

#### 3.3.1. Incremental Cycling Test (Mader Protocol)

**Figure 1:**
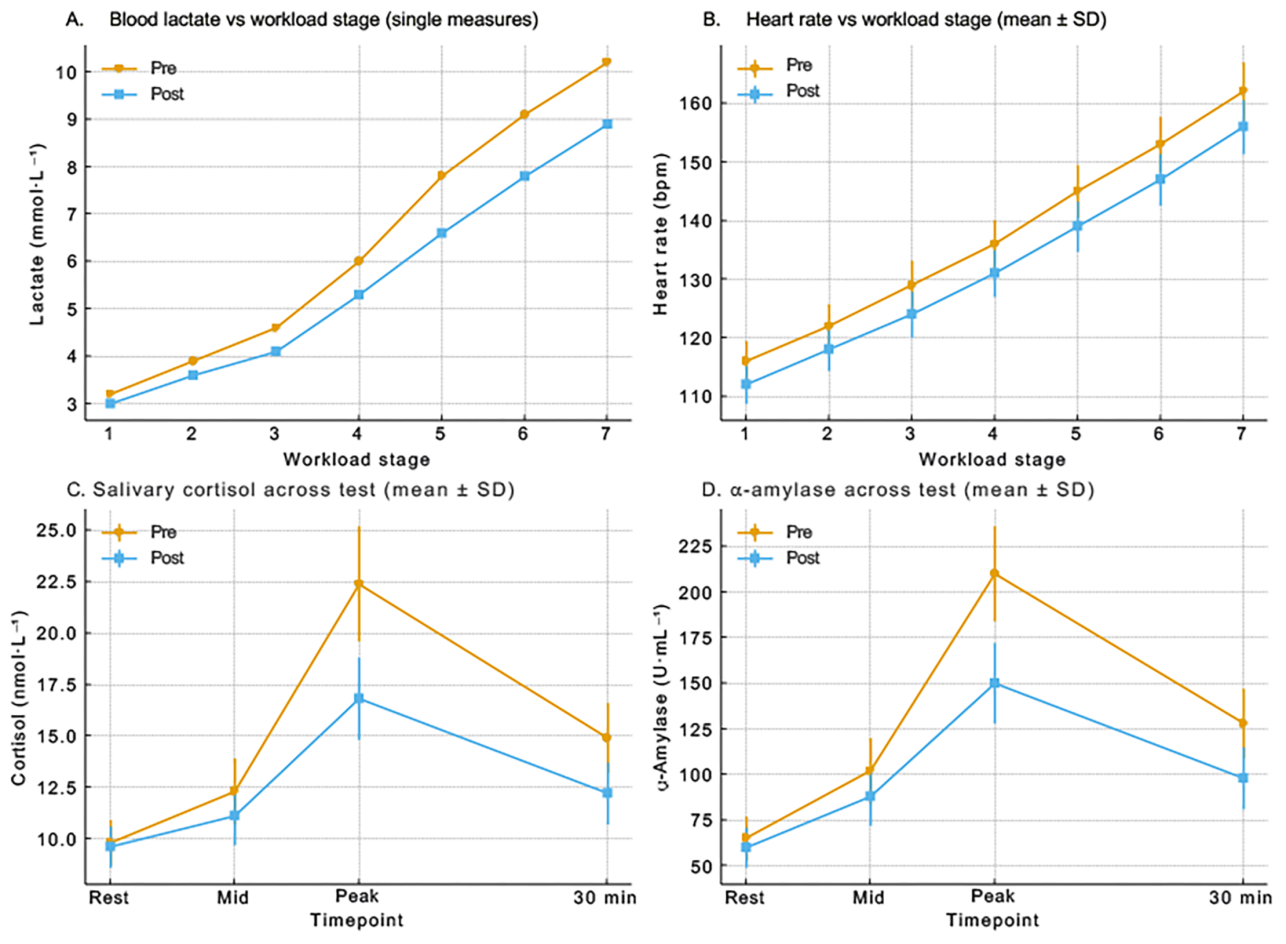
Physiological and biochemical responses to incremental exercise. Note. Lactate is shown as single end-of-stage values. Heart rate (HR) is presented as mean ± SD reflecting beat-to-beat variability during the final 30 s of each stage. Salivary biomarkers (sCort and sAA) are presented as mean ± SD representing analytical variability (technical error) of duplicate assays.

Post intervention, $\left[{\mathrm{La}}^{-}\right]$ concentrations were lower at all matched workloads compared to baseline. The peak lactate measured after the final stage decreased from 9.7 to 7.8 mmol·L^−1^ (−19.5%), while the power output at the 4 mmol·L^−1^ threshold (W4) shifted from 125 W to 145 W (+16.0%; Table 3; Figure 1). The final workload achieved increased by one stage (+25 W).

Heart rate, averaged over the final 30 s of each stage, was lower across the protocol after BHT. The mean HR recorded during the test sessions declined from 130 ± 5.8 bpm to 125 ± 5.1 bpm (−3.8%). A reduction in the HR area under the curve (AUC) was also observed.

#### 3.3.2. Pulmonary Function Testing (Spirometry and DLCO)

Spirometry: Pre- and post-BHT values remained within physiological norms. Recorded changes included a decrease in MEF_50_ and MEF_25_, and an increase in inspiratory vital capacity (VC IN) (Table 4).

DLCO Testing: $DLCO_{sb}$ increased from 9.53 to 10.01 mmol/min·kPa (+5.0%), with the Z-score rising from +1.66 to +2.03. KCO increased from 1.72 to 1.78 mmol/min·kPa·L. Hemoglobin concentration remained stable (pre: 13.1 g/dL; post: 13.1 g/dL).

#### 3.3.3. Cardiopulmonary Exercise Testing (CPET)

Post BHT, relative ${\mathrm{VO}}_{2\mathrm{peak}}$ increased by 3.8%, and oxygen pulse rose by 9.1%. The ${\mathrm{VE}/\mathrm{VCO}}_{2}$ slope decreased from 25.2 to 23.9, and ${\mathrm{EqCO}}_{2}$ decreased from 32.5 to 31.0.

#### 3.3.4. Salivary Biomarkers of Stress (sCort and sAA)

Post intervention, peak sCort declined from 22.4 ± 2.1 to 16.8 ± 1.9 nmol·L^−1^, while mean levels decreased from 13.2 ± 1.7 to 11.1 ± 1.4 nmol·L^−1^ (−15.9%). sAA mean values fell from 151.3 ± 18.5 to 110.0 ± 15.6 U·mL^−1^ (−27.3%).

### 3.4. Reliability and Measurement Uncertainty

To ensure clarity in interpreting the single-case data, HR and salivary biomarkers (sCort and sAA) are reported as mean ± standard deviation (SD) to reflect specific sources of variability. For HR, the SD represents the beat-to-beat variability recorded during the final 30 s of each exercise stage. For salivary markers, the SD reflects the analytical variability (technical error) between duplicate ELISA assays. Blood lactate ($\left[{\mathrm{La}}^{-}\right]$) and W4 power were derived from single-point measurements and interpolation according to the standard Mader protocol. Observed changes were evaluated against known coefficients of variation and technical errors of measurement to distinguish potential physiological signals from background noise.

## 4. Discussion

This signal-generating single-case study suggests that an eight-week BHT program elicited coordinated adaptations in metabolic, cardiovascular, pulmonary, and neuroendocrine systems during an incremental exercise test in a national-level swimmer. Notably, we observed a downward and rightward shift in the $\left[{\mathrm{La}}^{-}\right]$ curve, reduced HR at submaximal and maximal workloads, and attenuated sCort and sAA responses. These physiological changes were accompanied by an increase in the diffusing capacity of the $DLCO_{sb}$, reflecting a potential increase in pulmonary gas exchange efficiency, and by higher peak oxygen uptake (${\mathrm{VO}}_{2\mathrm{peak}}$) and oxygen pulse values, indicative of enhanced aerobic capacity and stroke volume efficiency. Collectively, these findings point toward improved metabolic economy, ventilatory and cardiovascular efficiency, and reduced physiological stress reactivity following BHT.

### 4.1. Metabolic Adaptations and Blood Lactate Accumulation

The observed downward and rightward shift in the $\left[La^{-}\right]$ curve, with a 16% increase in W4, aligns with evidence that apnea training can modulate metabolic thresholds [3,25]. However, since we did not directly measure lactate turnover, mitochondrial enzyme activity, or monocarboxylate transporter (MCT) expression, the underlying mechanisms remain speculative. These changes should be interpreted as a reduction in $\left[{\mathrm{La}}^{-}\right]$ accumulation during the specific test conditions rather than confirmed alterations in metabolic clearance or production rates [22,26].

Physiologically, a reduced lactate response at matched workloads is a plausible indicator of improved metabolic economy. In short-distance swimming (50–100 m), enhanced aerobic efficiency during the initial phases may preserve the anaerobic reserve for the final sprint, potentially mitigating neuromuscular fatigue. Furthermore, for a national-level sprinter, minimizing breathing frequency is a critical strategy to maintain hydrodynamic alignment. Each breath has been shown to potentially penalize velocity by approximately 0.02 s [20,21]. By potentially enhancing ${\mathrm{CO}}_{2}$ tolerance and metabolic efficiency through BHT, the athlete may reduce breathing frequency during 50 m and 100 m events, thereby minimizing kinematic disruptions. While these findings suggest that BHT provided a novel stimulus beyond standard training, the synergistic effect of the athlete’s high-volume swimming routine cannot be excluded as a contributing factor to these metabolic shifts.

### 4.2. Cardiovascular and Autonomic Modulation

Post-intervention HR reductions during incremental exercise support the hypothesis that BHT enhances autonomic balance via increased vagal tone and reduced sympathetic drive [19,27]. This interpretation is consistent with Seiler and Kjerland [28], who showed that endurance athletes often benefit from improved stroke volume and overall cardiac efficiency under hypoxic stress. The repeated activation of the diving reflex during BHT likely contributed to bradycardic adaptations [1,29], while peripheral chemoreceptor desensitization may have played a complementary role in modulating ventilatory and cardiovascular responses [30].

### 4.3. Neuroendocrine Response

The attenuated sCort and sAA responses observed post intervention may suggest a potential reduction in neuroendocrine reactivity to the exercise stimulus, rather than a definitive recalibration of the HPA axis and SAM system [11,17]. In national-level sport contexts, such blunting of stress reactivity has been associated with improved tolerance to training loads and more efficient recovery [13,31]. Our findings align with the hypothesis that repeated exposure to hypoxic–hypercapnic stress during BHT might condition the neuroendocrine system to respond more economically, which is consistent with evidence suggesting that chronic controlled exposure can dampen acute HPA reactivity [32].

These neuroendocrine observations are also compatible with broader systemic adaptations reported in intermittent hypoxic and apnea training research [7,33]. While our results regarding reduced peak sCort levels diverge from the transient elevations observed by Hooshmand et al. [34] under competitive stress, they suggest that structured BHT might favor a more efficient stress management strategy. However, given the single-case nature of this study, these shifts should be interpreted as suggestive physiological signals, as other confounding factors like seasonal training variations or the athlete’s recovery status could have influenced the observed biomarkers.

### 4.4. Pulmonary Function and Gas Exchange Efficiency

The comparison between pre- and post-BHT respiratory parameters suggests a potential shift in the athlete’s cardiorespiratory efficiency. Notably, the observed increase in $DLCO_{sb}$ (+5.0%) aligns with previous research suggesting that BHT may enhance pulmonary gas exchange efficiency [35,36]. However, it must be considered whether such a change exceeds the expected test–retest variability for single-breath diffusing capacity in elite athletes. While this increase is physiologically interesting, it should be viewed as a supportive observation rather than confirmed evidence of structural alveolar-capillary adaptations.

Spirometric indices showed general stability in volumes and flow rates, consistent with the notion that BHT primarily impacts diffusion capacity and cardiovascular efficiency rather than ventilatory mechanics [16]. Regarding CPET-derived values, the 9.1% rise in oxygen pulse is suggestive of potential improvements in aerobic capacity. Nevertheless, as oxygen pulse is an indirect index, these findings cannot definitively distinguish between increased stroke volume and enhanced peripheral oxygen extraction without direct invasive measurements. Concurrently, the improved ventilatory efficiency (reflected in the $VE/{\mathrm{VCO}}_{2}$ slope and ${\mathrm{EqCO}}_{2}$) may suggest a lower metabolic cost for a given workload and enhanced autonomic control, although these associations remain speculative in the context of a single-case design [37].

### 4.5. Individualization and Practical Implications

The subject’s national-level background and familiarity with swimming-related hypoxic exposure likely facilitated the robust adaptations observed, aligning with prior reports that responses to BHT vary based on fitness level and training history [4]. Furthermore, the improvements observed were likely facilitated by the individualization of workload and progressive adjustment of apnea tasks. This personalized approach ensured that the hypoxic–hypercapnic stimulus was delivered within tolerable yet progressively challenging limits, avoiding excessive fatigue while maintaining ecological validity [38,39].

Beyond the physiological findings, we propose a theoretical multimodal monitoring framework (Table 5). This protocol is derived from a synthesis of the current literature and our clinical observations; it is intended as a practical suggestion for coaches and should not be considered a validated outcome of the present case study.

### 4.6. Methodological Considerations and Limitations

A primary limitation of this single-case study is the inherent difficulty in isolating the specific effects of BHT from the athlete’s concomitant high-volume training program. As the subject was a national-level swimmer, the observed adaptations likely reflect a synergistic effect rather than an isolated response to apnea training alone. While the athlete was in a stable maintenance phase of her season, other confounding factors such as day-to-day biological variability, test familiarization, recovery status, nutrition, and sleep quality cannot be entirely excluded as potential contributors to the observed changes. Although testing was standardized for the early follicular phase of the menstrual cycle, the lack of a repeated-baseline design further limits the ability to establish definitive causality.

Furthermore, while cycle ergometry was chosen for pre/post testing to ensure standardized workload increments and reproducibility [25], this modality does not replicate the specific biomechanics of swimming; thus, the results should be interpreted as systemic physiological observations rather than exclusively sport-specific performance outcomes. The absence of a swimming-specific maximal performance test (e.g., a 50 m or 100 m sprint) also represents a limitation. While laboratory-based assessments allowed for precise control of workload and gas exchange ($DLCO_{sb}$), future studies should incorporate both laboratory and sport-specific trials to better correlate physiological signals with direct competitive performance. Consequently, these findings should be viewed as hypothesis-generating and preliminary, serving as a basis for future randomized controlled trials involving larger cohorts and control conditions to confirm the potential of BHT as an adjunct training stimulus.

## 5. Conclusions

This signal-generating single-case study suggests that an eight-week BHT program, integrated into a national-level swimmer’s regimen, can lead to favorable shifts in metabolic, cardiovascular, and neuroendocrine responses. The observed reductions in $\left[{\mathrm{La}}^{-}\right]$ accumulation and heart rate, alongside a blunted salivary stress biomarker profile and increased $DLCO_{sb}$, point toward a systemic adaptation to the hypoxic–hypercapnic stimulus.

The individualized and progressive nature of the BHT protocol was likely a key factor in ensuring these adaptations were achieved without adverse effects or excessive fatigue. While the single-case design and the “add-on” nature of the intervention preclude definitive causal inferences or the isolation of specific physiological mechanisms (e.g., lactate clearance vs. production), the results provide a strong rationale for the use of BHT as a complementary training stimulus in elite aquatic sports.

From a practical standpoint, this study highlights the potential of structured BHT to enhance metabolic economy and stress resilience. Future research should prioritize randomized controlled trials with larger cohorts and swimming-specific performance tests to confirm these preliminary findings and further explore the underlying molecular and ventilatory pathways of adaptation.

## Figures and Tables

**Table 1:** Anthropometric, hematological, and biochemical profile of the national-level swimmer.

Parameter	Value	Ref. Range (Female Athletes)
Age (years)	23	—
Height (m)	1.72	—
Weight (kg)	63.0	—
BMI (kg/m^2^)	22.9	18.5–24.9
Phase angle (°)	7.0	5.0–9.0
Resistance (Ω)	561	250–600
Reactance (Ω)	69	30–80
Total body water (%)	52.3	55–65
Fat-free mass (%)	75.2	75–90
Body fat (%)	24.8	12–22
Hemoglobin (g·dL^−1^)	13.1	12–16
Hematocrit (%)	37.1	35–48
WBC (×10^3^ μL^−1^)	4.83	3.8–10
C-reactive protein (CRP) (mg·dL^−1^)	0.01	0–0.5
TSH (μIU/mL)	0.24	0.1–1.0
Albumin (g·dL^−1^)	4.11	3.5–5.2

Note. Values were obtained at baseline using standardized body composition analysis procedures.

**Table 2:** Performance metrics and functional benchmarks of the national-level swimmer.

Parameter	Value
Best 50 m freestyle time (s)	26.6
Best 100 m freestyle time (s)	60.4
Training frequency (sessions·wk^−1^)	13
Training volume (km·wk^−1^)	>20
Specialization	Short-distance freestyle (50–100 m)

Note. Swimming times were measured in a 50 m Olympic pool under official competition conditions.

**Table 3:** Summary of key physiological and neuroendocrine observations following BHT. Values for HR and salivary biomarkers represent measurement variability (intra-stage SD and analytical SD, respectively).

Variable	Pre-BHT	Post-BHT	% Change
${\left[La^{-}\right]}_{max}$ (mmol·L^−1^)	9.7	7.8	−19.5
Heart rate (bpm)	130 ± 5.8 ^a^	125 ± 5.1 ^a^	−3.8
Salivary cortisol (nmol·L^−1^)	13.2 ± 2.4 ^b^	11.1 ± 1.9 ^b^	−15.9
Salivary α-amylase (U·mL^−1^)	151.3 ± 28.1 ^b^	110.0 ± 22.0 ^b^	−27.3
W4 workload (W)	125	145	+16.0

^a^ SD represents heart rate variability during the final 30 s of the exercise stage; ^b^ SD represents duplicate assay variability (technical error).

**Table 4:** Spirometry, $DLCO_{sb}$, and CPET parameters: observed values pre- vs. post BHT.

Parameter	Pre-BHT	Post-BHT	% Change
FEV1 (L)	3.12	3.09	−0.96
FVC (L)	3.96	3.93	−0.76
VC IN (L)	3.88	4.10	5.67
$DLCO_{sb}$ (mmol·min^−1^·kPa^−1^)	9.53	10.01	5.04
KCO (mmol·min^−1^·kPa^−1^·L^−1^)	1.72	1.78	3.49
${\mathrm{VO}}_{2\mathrm{peak}}$ (mL·kg^−1^·min^−1^)	45.1	46.8	3.77
O_2_ Pulse (mL·beat^−1^)	16.5	18.0	9.09
Peak HR (bpm)	186	181	−2.69
VE/VCO_2_ slope	25.2	23.9	−5.16
EqCO_2_	32.5	31.0	−4.62

**Table 5:** Proposed monitoring protocol to support safe implementation of BHT in national-level athletes.

Domain	Measure	Rationale	Frequency
Metabolic	Lactate kinetics (incremental or submaximal test)	Tracks metabolic efficiency, detects abnormal lactate accumulation patterns	Every 2–4 weeks
Cardiovascular	Resting HR and HRV (morning, supine)	Evaluates autonomic balance; reduced HRV may signal maladaptation	Daily or weekly trend
Neuroendocrine	Salivary cortisol and α-amylase	Non-invasive stress biomarkers; blunted or exaggerated responses indicate dysregulation	Pre/post key training cycles
Perceptual	Session-RPE, fatigue and recovery scales	Captures subjective training load and readiness to complement physiological data	Every session
Training diary	Workload, apnea duration, recovery intervals	Ensures accurate documentation and supports progressive, individualised load	Every session

## Data Availability

The data presented in this study are available on request from the corresponding author. The data are not publicly available, due to privacy restrictions regarding the identity of the national-level athlete involved.

## Supplementary Materials

The following supporting information can be downloaded at https://www.mdpi.com/article/10.3390/jfmk11020213/s1, File S1: Detailed 8-week Breath-Hold Training (BHT) Protocol.
