# Supplementary material for: Metabolic, Cardiovascular, and Stress Biomarker Adaptations to Breath-Hold Training in a National-Level Swimmer: A Signal-Generating Single-Case Study
Source: J Funct Morphol Kinesiol. 2026 May 28;11(2):213. doi: 10.3390/jfmk11020213 (PMC13301196; doi:10.3390/jfmk11020213)
Supplement: Supplementary file 1 [file jfmk-11-00213-s001.zip › jfmk-4300488-supplementary.pdf]

Supplementary Material: Detailed 8-Week Breath-Hold Training (BHT) Protocol

This document provides a comprehensive description of the 8-week Breath-Hold Training (BHT) intervention integrated into the athlete's standard swimming regimen. The protocol was designed to enhance CO_2 tolerance, hypoxic buffering capacity, and autonomic efficiency.

1. General Framework

- **Duration:** 8 consecutive weeks.
- **Frequency:** 3 supervised sessions per week (Monday, Wednesday, Friday).
- **Modality:** Combined dry-land static apnea and in-water dynamic apnea sessions.
- **Supervision:** All sessions conducted by certified swimming and freediving coaches.
- **Safety:** Adherence to International safety guidelines for apnea (Lindholm, 2009).

2. Dry-Land Protocol (Static Apnea)

Performed in a supine position before the swimming session. The focus was on maximal duration while maintaining relaxation.

Component	Details
**Lung Volume**	Total Lung Capacity (TLC) - Full inspiration.
**Volume**	3 sets of 5 maximal voluntary apneas.
**Recovery**	2 minutes passive recovery between apneas; 5 minutes between sets.
**Progression**	+5% total breath-hold volume every 2 weeks based on RPE < 7/10.

3. In-Water Protocol (Dynamic Apnea)

Integrated into the specific swimming endurance block. Performed in a 25m or 50m pool.

Week	Repetitions	Breathing Pattern	UW Dolphin Kick	Recovery
1-2	8 x 50m	Every 5 strokes	15 m	60 s
3-4	8 x 50m	Every 7 strokes	15 m	45 s
5-6	8 x 50m	Every 9 strokes	20 m	30 s
7-8	8 x 50m	Every 9 strokes	25 m	30 s

Note: All repetitions performed at ~70% of maximal Heart Rate (HR).

4. Monitoring and Load Adjustment

- **Weekly Diary:** The athlete recorded perceived exertion (RPE), sleep quality, and physical readiness.
- **Heart Rate:** Monitored during all water sessions via waterproof HR sensor.
- **Adjustment Criteria:** Progression was delayed if RPE was > 8/10 for two consecutive sessions or if HR recovery between repetitions was significantly impaired.
